# The impact of diabetes prevention on labour force participation and income of older Australians: an economic study

**DOI:** 10.1186/1471-2458-12-16

**Published:** 2012-01-06

**Authors:** Megan E Passey, Rupendra N Shrestha, Melanie Y Bertram, Deborah J Schofield, Theo Vos, Emily J Callander, Richard Percival, Simon J Kelly

**Affiliations:** 1 University Centre for Rural Health--North Coast, School of Public Health, University of Sydney, 61 Uralba St, Lismore, NSW 2480, Australia; 2 NHMRC Clinical Trials Centre, University of Sydney, 92-94 Parramatta Rd, Camperdown, NSW 1450, Australia; 3 School of Population Health, University of Queensland, Herston Rd, Herston, QLD 4006, Australia; 4 Sydney School of Public Health, University of Sydney, Camperdown, NSW 1450, Australia; 5 National Centre for Social and Economic Modelling, University of Canberra, University Drive South, Bruce, ACT 2601, Australia

## Abstract

**Background:**

Globally, diabetes is estimated to affect 246 million people and is increasing. In Australia diabetes has been made a national health priority. While the direct costs of treating diabetes are substantial, and rising, the indirect costs are considered greater. There is evidence that interventions to prevent diabetes are effective, and cost-effective, but the impact on labour force participation and income has not been assessed. In this study we quantify the potential impact of implementing a diabetes prevention program, using screening and either metformin or a lifestyle intervention on individual economic outcomes of pre-diabetic Australians aged 45-64.

**Methods:**

The output of an epidemiological microsimulation model of the reduction in prevalence of diabetes from a lifestyle or metformin intervention, and another microsimulation model, Health&WealthMOD, of health and the associated impacts on labour force participation, personal income, savings, government revenue and expenditure were used to quantify the estimated outcomes of the two interventions.

**Results:**

An additional 753 person years in the labour force would have been achieved from 1993 to 2003 for the male cohort aged 60-64 years in 2003, if a lifestyle intervention had been introduced in 1983; with 890 person years for the equivalent female group. The impact on labour force participation was lower for the metformin intervention, and increased with age for both interventions. The male cohort aged 60-64 years in 2003 would have earned an additional $30 million in income with the metformin intervention, and the equivalent female cohort would have earned an additional $25 million. If the lifestyle intervention was introduced, the same male and female cohorts would have earned an additional $34 million and $28 million respectively from 1993 to 2003. For the individuals involved, on average, males would have earned an additional $44,600 per year and females an additional $31,800 per year, if they had continued to work as a result of preventing diabetes.

**Conclusions:**

In addition to improved health and wellbeing, considerable benefits to individuals, in terms of both additional working years and increased personal income, could be made by introducing either a lifestyle or metformin intervention to prevent diabetes.

## Background

Globally, diabetes is estimated to affect 246 million people (2007 figure), with this figure expected to reach 380 million by 2025 [1]. The increase has been attributed to increases in obesity and sedentary lifestyles, and the ageing of the global population [2,3]. In Australia, the prevalence of obesity has increased from 43% of the population aged 15 and over in 1995 to 60% in 2007-08 [4] and has been identified as the largest contributor to the burden of disease associated with diabetes [5]. Diabetes has been made a national health priority area in recognition of the personal and public costs of the disease [6]. The cost of treating diabetes is expected to rise by 436% (or $7 billion, in Australian currency) from 2003 to 2033 [7]. However, while the direct costs of treating diabetes are expected to rise dramatically the indirect costs are considered greater [2].

The indirect costs are mostly attributed to lost workforce participation and productivity [2,8]. Within Australia 37.9% of people aged 45-64 who identify diabetes as their main health condition are not in the labour force, compared with only 30% of all those aged 45-64 [9]. People who retire early due to diabetes are less likely to have any financial assets (such as superannuation, investment properties and equity in their own homes), and if they do, the value of their assets is around 90% less, than those who remain in the labour force full time with no chronic health problem [10]. Other studies have identified the significant costs that lost income following early retirement due to poor health have at the national level [11].

People with impaired fasting glucose or impaired glucose tolerance are at high risk of developing type 2 diabetes and are considered pre-diabetic [12]. Several studies have demonstrated that interventions preventing (or delaying) the development of type 2 diabetes in high risk individuals are effective [13-15]. Recently, Bertram *et al. *investigated the health impacts and health costs of a number of interventions to prevent diabetes. They concluded that screening to identify people with pre-diabetes, followed by treatment with metformin or diet and exercise for those at risk were the most cost-effective interventions in preventing or delaying the onset of diabetes [16]. In addition to health benefits, prevention of diabetes would reduce the costs associated with early retirement due to diabetes.

This study will build upon the findings of Bertram *et al. *and estimate the impact on labour force participation and personal income of a diabetes prevention intervention using screening and treatment (metformin or a lifestyle intervention targeting diet and exercise) in pre-diabetic Australians aged 45-64. The age group of 45-64 year olds is considered particularly important as this age group has a high rate of early retirement and will make up an increasing proportion of the working population as Australia's population ages [17].

## Methods

We estimated the extra number of years in the labour force of people aged 45-64 years in 2003 who would not have developed diabetes if a screening and intervention program to prevent the onset of diabetes were in place for the 20 years from 1983. We further estimated the potential increase in their personal incomes as a result of staying in the workforce.

### Diabetes and labour force participation

Prevalence of diabetes estimates were based on the Australian Bureau of Statistics (ABS) Surveys of Disability, Ageing and Carers (SDAC) conducted in 1993, 1998 and 2003. Chronic health conditions were self reported in the surveys and were grouped according to the ABS classification of chronic health conditions which were based on International Classification of Diseases, Version 10 (ICD-10) codes for the 1998 and 2003 surveys and ICD-9 codes for the 1993 survey. The prevalence of self reported diabetes was estimated for each year from 1993 to 2003 for each 5 year age group of the baseline population aged 25 to 44 years old in 1983. For non-survey years, the prevalence of self reported diabetes and the total population were first estimated using linear interpolation from the two nearest surveys and then the number of self reported diabetes cases was estimated. The prevalence of self-reported diabetes was not estimated before 1993 as there were no SDAC data available prior to this time.

Using the 2003 Survey of Disability, Ageing and Carers, differences in probabilities of the labour force participation of those without diabetes and of those with diabetes were estimated for each 5 year age and gender group, adjusted for highest level of education attained. Labour force participation was defined as being either employed or looking for work. People who were neither employed nor looking for work were categorised as "out of the labour force".

In order to estimate additional income that would have been accumulated had the screening and intervention been in place since 1983, we derived income estimates of the individuals from *Health&WealthMOD*, which is Australia's first microsimulation model of health and disability, the associated impacts on labour force participation, personal income, savings, government revenue and expenditure [18]. This model was specifically designed to measure the economic impacts of ill health on Australian workers aged 45 to 64 years; information which was previously unavailable.

The base population of Health&WealthMOD was unit record data extracted from the Survey of Disability, Ageing and Carers conducted by the Australian Bureau of Statistics in 2003 [19]. From this dataset, individual records were extracted for those aged 45-64 years. The details extracted for each individual in the base population included demographic variables (for example, age, sex, family type, state of residence, and ethnic background), socioeconomic variables (level and field of education, income, benefits received), labour force variables (labour force participation, employment restrictions, retirement), and health and disability variables (chronic conditions, health status, type and extent of disability, support and care required).

Using a separate microsimulation model--STINMOD--additional economic information such as individual income, government support payments and tax liability was imputed onto the base data. STINMOD is Australia's leading model of income tax and government support payments [20,21], and is maintained and developed for the Australian Government by the National Centre for Social and Economic Modelling. Income and wealth information was imputed onto the base population of Health&WealthMOD by identifying persons with similar characteristics on STINMOD and "donating" their income and wealth information onto Health&WealthMOD using a process commonly used in microsimulation modelling called synthetic matching [22]. Nine variables: sex (2 groups), income unit type (4 groups), type of government pension/support (3 groups), income quintile (5 groups), age group (4 groups), labour force status (4 groups), hours worked per week (5 groups), highest educational qualification (2 groups) and home ownership (2 groups), that were common to both datasets and strongly related to income were chosen as matching variables for synthetic matching.

The data were then aged to reflect the 2009 Australian 45 to 64 year old population. The up-rating was used to account for the disability and illness, demographic, labour force, earnings growth and other changes that had occurred between 2003 and 2009.

Income estimates from Health&WealthMOD, which were in 2009 dollars, were converted to 2003 dollars by adjusting for the change in consumer price indices from 2003 to 2009. Difference between median incomes, adjusted for age group and highest education, of those who were in the labour force and had no diabetes, and of those who were not in the labour force and had diabetes was considered as an estimated additional income an individual would have if the person did not develop diabetes because of the intervention and remained in the labour force.

### Reduction in the prevalence of diabetes due to interventions

An epidemiological microsimulation model was used to predict the percentage reduction in the prevalence of biochemically confirmed diabetes that would have been achieved if a screening program for pre-diabetes and metformin or lifestyle intervention for those identified with pre-diabetes had been in place for the twenty years starting from 1983. Full details of the model are available elsewhere [16]. Briefly, it is a discrete time microsimulation model which models the 2003 cohort of Australian people free of diabetes. These people may have normal glucose tolerance or pre-diabetes. This model allows people to progress from normal glucose tolerance to pre-diabetes and then diabetes. Progression through disease states is governed by epidemiological data from the Australian Burden of Disease and Injury study and from a study on the epidemiology of pre-diabetes [5,23]. These disease parameters were back cast to 1983 to enable the screening program to be modelled for 20 years. People who are aged 45 or over with one or more risk factors for diabetes, (including overweight or obesity, family history of type 2 diabetes and previous gestational diabetes); who are Indigenous Australians; or who are over 55 years of age were eligible for a screening program to identify pre-diabetes. We modelled 65% of GPs participating in a screening program, based on the number of GPs who participate in the current practice incentive program for diabetes [24]. We then assumed that they would ask 80% of those eligible for screening to participate and that 70% of these people would agree to participate in screening (based on a National Heart Foundation consensus panel) [25]. We assumed that, if identified as pre-diabetic, they would then be treated with either metformin or a lifestyle change intervention addressing diet and exercise. Reductions in diabetes prevalence were predicted for every year from 1984 to 2003 for each 5 year age group of the population, who were 25 to 44 years old in 1983 and who would have been 45 to 64 years old in 2003.

Although the epidemiological microsimulation model predicted the percentage reduction in the prevalence of diabetes due to interventions from 1983 to 2003, we only estimated the impacts of interventions on increased labour force participation and additional incomes from 1993 to 2003. This is for two reasons. The first reason is the lack of survey data, on which we based our estimates of diabetes prevalence, for the years prior to 1993. SDAC data were available only for the three last surveys conducted in 1993, 1998 and 2003. The second reason is that a large proportion of the baseline population of 25 to 44 years old are not eligible for screening for pre-diabetes as one needs to be 45 years or over to become eligible; the age cohorts gradually became eligible through the period 1983-2003.

### Assumptions

In order to model additional person years in the labour force and consequently additional income as a result of the screening program for pre-diabetes and the intervention for those identified as pre-diabetic for the twenty years starting from 1983, the following assumptions are made:

1. The percentage reduction in the prevalence of biochemically confirmed diabetes each year because of the screening and intervention program are the same as the percentage reduction in the prevalence of self-reported diabetes for the respective years.

2. Differences in the labour force participation rates, adjusted for highest education, of those with no diabetes and with diabetes have not changed over the years.

### Simulation

We first estimated the reduction in the number of self-reported diabetes cases (i.e. extra number of diabetes free persons) in each year from 1993 to 2003 for each 5 year age group by applying the percentage reduction in biochemically confirmed diabetes prevalence for the respective age cohort to the number of self reported diabetes cases in each year for the same age cohort. The number of additional persons in the labour force each year was then calculated by applying age group specific differences in the probability of labour force participation of those without diabetes and of those with diabetes to the extra number of diabetes free persons of the same age group. For example, for the cohort who were 60 to 64 years old in 2003, the extra number of diabetes free persons in 1993, 1998 and 2003 were respectively multiplied by the differences in the probability for age groups 50 to 54, 55 to 59 and 60 to 64 years to estimate the extra number of persons in the labour force in 1993, 1998 and 2003 for this cohort due to the screening program and interventions. The total extra person years in the labour force from 1993 to 2003 for each cohort was then multiplied by the adjusted difference in median incomes of those who were in the labour force and had no diabetes, and of those who were not in the labour force and had diabetes to estimate the additional income (in 2003 dollars) that would have been accumulated by each cohort over the ten years from 1993 to 2003. In order to simulate the results for the entire Australian population of age group 45 to 64 years, we performed a weighted analysis using the weights assigned by the ABS to each survey record which represented the number of similar individuals in the Australian population. All modelling was done separately for males and females, and for the two interventions; lifestyle and pharmaceutical intervention using metformin.

Ethical approval was not required, but the research conformed to the Helsinki Declaration http://www.wma.net/en/30publications/10policies/b3/, and to local Australian legislation. The base data used for this modelling came from the Australian Bureau of Statistics and are available to researchers on request.

## Results

**Figure 1:**
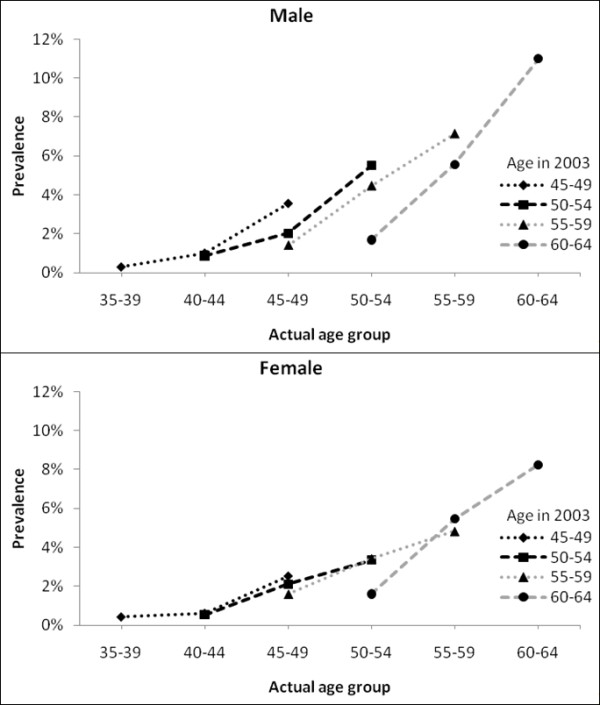
**Prevalence of diabetes by age-sex cohort**.

The likelihood of developing diabetes increases with age (Figure 1) and over time, with an increasing trend in the prevalence of diabetes within each age group. The prevalence of diabetes for males aged 50-54 years old is 1.68% in 1993 compared to 4.46% in 1998 and 5.51% in 2003. Although the prevalence is slightly lower for females compared to males, the trends in prevalence are similar for both genders.

Table 1 shows the reduction in the prevalence of diabetes due to metformin or lifestyle interventions. The effects of the interventions in reducing the prevalence of diabetes are nil in the years prior to 1993 (not shown in Table 1), except for the male cohort aged 40 to 44 years old at baseline, for whom the reduction in prevalence started in 1987 (not shown in Table 1). Thus, our analysis restricted to the years from 1993 to 2003 estimates all of the impacts of the interventions except for the impacts on the male cohort, who were 40 to 44 years old at baseline.

**Table 1:** Percentage reduction in the prevalence of biochemically confirmed diabetes due to interventions

Age group at baseline (in 1983)		Year										
		
		1993	1994	1995	1996	1997	1998	1999	2000	2001	2002	2003
**Pharmaceutical intervention using metformin**												

Male	25-29	0	0	0	0	0	0	0	0	0	0.42	0.41
	
	30-34	0	0	0	0	0	0	0	0.43	0.63	0.83	0.96
	
	35-39	0	0.24	0.23	0.23	0.30	0.45	0.44	0.86	1.41	1.36	1.65
	
	40-44	0.64	0.94	1.24	1.53	1.45	1.65	1.85	2.15	2.43	2.07	2.13

Female	25-29	0	0	0	0	0	0	0	0	0	0.28	0.27
	
	30-34	0	0	0	0	0	0	0	0	0	0.41	0.40
	
	35-39	0.35	0.34	0.66	1.29	1.14	1.12	1.32	1.29	1.46	1.40	1.53
	
	40-44	0.49	0.48	1.18	1.63	1.20	1.34	1.48	1.62	2.07	1.69	1.90

**Lifestyle intervention**												

Male	25-29	0	0	0	0	0	0	0	0	0	0.62	0.61
	
	30-34	0	0	0	0	0	0	0.22	0.64	0.85	0.97	1.09
	
	35-39	0.24	0.48	0.47	0.46	0.45	0.60	0.73	1.01	1.84	1.67	1.75
	
	40-44	0.64	1.10	1.24	1.53	1.45	1.65	2.06	2.15	2.86	2.40	2.46

Female	25-29	0	0	0	0	0	0	0	0	0	0.28	0.27
	
	30-34	0	0	0	0	0	0	0	0	0	0.41	0.40
	
	35-39	0.35	0.68	0.99	1.62	1.37	1.35	1.54	1.50	1.67	1.71	1.84
	
	40-44	0.49	0.48	1.18	1.63	1.37	1.51	1.81	1.94	2.38	1.94	2.02

People who have diabetes have less probability of being in the labour force than those who do not have diabetes for all age groups. While the differences in these probabilities are similar for all male age groups between 40 and 64 years, they are dependent upon age for females, with females aged 50-54 and 55-59 years having larger difference than females aged 45-49 and 60-64 years old (Table 2).

**Table 2:** Labour force participation rates of people without diabetes and with diabetes and with differences in participation rates adjusted for highest education level, SDAC 2003

		Without diabetes	With diabetes	Difference^#^
Male	40-44	0.93	0.81	0.10
	45-49	0.90	0.74	0.16
	50-54	0.88	0.78	0.10
	55-59	0.77	0.64	0.12
	60-64	0.59	0.45	0.13
Female	40-44	0.77	0.43	0.30
	45-49	0.79	0.69	0.10
	50-54	0.70	0.43	0.27
	55-59	0.52	0.21	0.33
	60-64	0.30	0.22	0.09

^#^Differences were adjusted for highest education attained

The results in Table 3 show the outcomes that could have been achieved over the ten years from 1993 to 2003 if the screening program for pre-diabetes and either a metformin or lifestyle intervention were adopted in 1983 to prevent diabetes developing. For males who were aged 60-64 years in 2003, if a metformin intervention had been introduced in 1983, this age group cumulatively would have an additional 683 person years in the labour force due to the prevention of diabetes. For the same male age group, if a lifestyle intervention had been introduced in 1983, by 2003 this group would have had an additional 753 person years in the labour force due to diabetes prevention. The impact on labour force participation is slightly less for females of the same age group, and increases with age, as the interventions prevent more people developing diabetes in older age.

**Table 3:** Increased number of person years in the labour force & the associated increased in total incomes over the ten years from 1993 to 2003 due to the interventions

	Age group in 2003	Over ten years	
		
		Total person years	Total incomes (2003 dollars)
***For pharmaceutical intervention using metformin***			
Male	45-49	28	1,263,000
	50-54	97	4,319,000
	55-59	282	12,578,000
	60-64	683	30,486,000

Female	45-49	11	347,000
	50-54	42	1,329,000
	55-59	679	21,629,000
	60-64	790	25,144,000

Total		2,612	97,095,000

***For lifestyle intervention***			
Male	45-49	43	1,896,000
	50-54	125	5,595,000
	55-59	358	15,967,000
	60-64	753	33,599,000

Female	45-49	11	347,000
	50-54	42	1,329,000
	55-59	816	25,983,000
	60-64	890	28,334,000

Total		3,038	113,049,000

The lifestyle intervention has higher impacts than the pharmaceutical intervention of using metformin, with the lifestyle intervention giving an additional 3,038 person years in the labour force compared to 2,612 person years due to the metformin intervention over the same ten year period.

As labour force participation would have been higher with either of the interventions, so too would the incomes produced. There would have been an additional $44,600 per man and $31,800 per woman on average for each year if they had continued to work as a result of preventing the onset of diabetes. An additional $30 million in income would have been achieved over the ten year period from 1993 to 2003 by the male cohort, who were 60-64 years old in 2003 if the metformin intervention was introduced in 1983, and an additional $25 million for the same time period for the equivalent female cohort (Table 3). If the lifestyle intervention was introduced in 1983, the same male and female cohorts would have achieved an additional $34 million and $28 million respectively in income over the ten year period from 1993 to 2003. For all cohorts, the metformin intervention would have resulted in an additional $97 million in income, and the lifestyle intervention in an additional $113 million.

## Discussion

In this paper we have estimated the potential impact of diabetes prevention programs on labour force participation and income generation among people at high risk of developing diabetes. The results show that considerable benefits, in terms of both additional working years and increased personal income, could be made by introducing either a lifestyle or metformin intervention to prevent diabetes. Up to an additional $113 million in total personal income between the years 1993 and 2003 could have been generated if a lifestyle intervention was implemented in 1983. While this amount is small relative to total income for this age group (income for this age group was $172 billion in 2003--results not shown), for the individuals involved, the impact is considerable.

Our results should be considered a minimal estimate of the impact of diabetes prevention interventions due to the conservative approach taken. Firstly, we have only captured the lost working years and income among those who retire. Among those who continue in the workforce, diabetes is likely to further reduce productivity through a shift to part-time or lower paid work as a result of the illness and its complications [26]. Indeed Schofield *et al. *have shown that those with diabetes have significantly lower incomes than those with no long term health condition regardless of labour force status [11]. Secondly, the assumptions used for estimating reductions in prevalence were based on conservative levels of participation in the screening program by both GPs and consumers. If participation rates could be increased, there would be greater reductions in the incidence of diabetes with consequent increases in workforce participation. Thirdly, in the case of the lifestyle intervention there are likely to be additional benefits through reductions in obesity and other diseases such as cardiovascular disease and cancer, which have not been considered in our modelling.

A limitation of the approach taken is that we have assumed that the reduction in self-reported prevalence of diabetes from a diabetes intervention would be similar to the reduction in biochemically confirmed diabetes. Although this seems plausible, it has not been validated. A further limitation is that our approach implies a causal relationship between having diabetes and the reduced rate of labour force participation among those with diabetes, relative to those without. It is possible that some other factor is confounding the relationship and may be reducing the labour force participation of those with diabetes. Diabetes, as well as risk factors for diabetes (such as obesity, physical inactivity and smoking) are more common in lower socio-economic groups [4]. By controlling for education level in the analysis, we aimed to minimise the potential for confounding by other socio-economic factors, or by other diseases which are also prevalent among lower socio-economic groups. However, the possibility of confounding by unknown factors cannot be excluded. Additionally, the possibility of reverse causality cannot be completely excluded--that is, that early retirement has led to development of diabetes. However, the French GAZEL study provides interesting results which suggest this is unlikely. This longitudinal, repeat-measures study over 15 years has found that having diabetes increases the risk of transition from employment to disability and retirement [27], but that retirement did not change the risk of developing a number of chronic diseases, including diabetes [28].

Several other studies have estimated the indirect cost of diabetes attributed to lost labour force participation, both through temporary absenteeism and permanent retirement due to ill health [29-32]. A US study estimated lost productivity costs of US$2.6 billion due to diabetes related absenteeism from work, and US $7.9 billion due to unemployment from diabetes related disability in 2007 [33]. Similarly, the indirect costs of diabetes were estimated to be 70.1 million EURO in Norway in 2005 [31]. Studies that have analysed the indirect costs of diabetes have mainly used the human capital approach [34], which is consistent with the approach we have taken in our study. However, these previous studies assessed the cost of illness, and did not consider the financial benefits of treatment. Our study estimates the potential productivity benefits of interventions to prevent diabetes using a microsimulation model to estimate the reduction in the prevalence of diabetes likely to be achieved through these interventions and linking this to models of the impact of diabetes on labour force participation.

The benefit of increased labour force participation as a result of preventing diabetes is likely to extend beyond the individual, and provide some medium-term benefit to government also. Previous studies have shown that in the 45 to 64 year age group diabetics pay 67% less tax than non-diabetics, and receive 112% more in social security payments each week than non-diabetics [11]. This is in addition to the $989 million that the Australian government allocates to health expenditure for diabetes (based on figures for the 2004-05 period) and the additional $7 billion that is projected to be spent between 2003 and 2033 [7,35]. Preventing diabetes is therefore likely to have considerable financial benefits to governments in the medium-term. While, the future costs to government of caring for subsequent illness among people whose death has been delayed are not considered in this analysis, it is the accepted responsibility of government to optimise the wellbeing of the population [36,37].

In the future, the benefits of diabetes prevention programs are likely to be greater, as diabetes is both increasing in prevalence worldwide, and becoming more common among the working-age population, particularly those aged 45 to 64 years [38]. Keeping greater numbers of experienced, older workers in the labour force is increasingly becoming a priority of governments. Key government reports have highlighted population ageing and labour shortages as potential pressures threatening the Australian economy [17]. Such a situation is likely to be reflected internationally in most developed countries, with the ageing of the global population [39].

The modelling undertaken assumes that the individuals who benefit from diabetes prevention would participate in the labour force at the same rate as other individuals without diabetes, controlling for education. Unlike many industrialised countries, Australia has low unemployment and labour shortages in a number of industries. In 2009, Australia's unemployment rate was 5.8%--close to the accepted 'full employment' rate of 5% [40]. In his 2011 budget speech, the treasurer emphasised the high employment rate: "Over 300,000 jobs have been created in the past year and the unemployment rate is forecast to fall further, to 4 1/2 per cent by mid 2013, creating another half a million jobs.... We believe our economy can't afford to waste a single pair of capable hands." [41] It is therefore likely that those who benefit from diabetes prevention would be able to find employment as easily as others.

Early retirement due to ill health is a problem throughout the world, and in Australia it is estimated that 58% of men and 26% of women who retire from full-time work early (that is, before the age of 55 years--from 55 years of age Australian citizens can access preserved superannuation and are entitled to some social security pensions) do so because of ill health [42]. Maintaining the health of the workforce is seen as a vital step in securing the economic activity of the nation [17]. The government has promoted deferred or gradual retirement as a solution, and given the numbers retiring due to illness, the prevention and treatment of long-term health conditions may be critical in helping older Australians to remain in the workforce longer [42]. There is also likely to be a widening gap between Australians who are able to work and those who retire prematurely when they become ill, in terms of both income earned and assets accumulated.

## Conclusions

This study suggests that prevention of diabetes with metformin or lifestyle interventions will have significant benefits for boosting labour force participation, and also increasing incomes for individuals.

## Competing interests

The authors declare that they have no competing interests.

## Authors' contributions

MP led the study, and along with DS conceived the original study idea. RS and MB undertook the modelling, and RS generated the results. All authors provided expert input to the design of the study and the interpretation of the results. EC drafted the manuscript and all authors contributed to its editing and have read and approved the final submission.

## Pre-publication history

The pre-publication history for this paper can be accessed here:

http://www.biomedcentral.com/1471-2458/12/16/prepub
